# Claudin-2 and claudin-12 form independent, complementary pores required to maintain calcium homeostasis

**DOI:** 10.1073/pnas.2111247118

**Published:** 2021-11-22

**Authors:** Megan R. Beggs, Kennedi Young, Wanling Pan, Debbie D. O’Neill, Matthew Saurette, Allein Plain, Juraj Rievaj, Michael R. Doschak, Emmanuelle Cordat, Henrik Dimke, R. Todd Alexander

**Affiliations:** ^a^Department of Physiology, University of Alberta, Edmonton, AB, T6G 2H7, Canada;; ^b^Women’s and Children’s Health Research Institute, Edmonton, AB, T6G 1C9, Canada;; ^c^Faculty of Pharmacy & Pharmaceutical Sciences, University of Alberta, Edmonton, AB, T6G 2H5, Canada;; ^d^Department of Cardiovascular and Renal Research, Institute of Molecular Medicine, University of Southern Denmark, 5000 Odense, Denmark;; ^e^Department of Nephrology, Odense University Hospital, 5000 Odense, Denmark;; ^f^Department of Pediatrics, University of Alberta, Edmonton, AB, T6G 1C9, Canada

**Keywords:** claudins, calcium, paracellular

## Abstract

Calcium (Ca^2+^) homeostasis is maintained through coordination between intestinal absorption, renal reabsorption, and bone remodeling. Intestinal and renal (re)absorption occurs via transcellular and paracellular pathways. The latter contributes the bulk of (re)absorption under conditions of adequate intake. Epithelial paracellular permeability is conferred by tight-junction proteins called claudins. However, the molecular identity of the paracellular Ca^2+^ pore remains to be delineated. Claudins (*Cldn*)-2 and -12 confer Ca^2+^ permeability, but deletion of either claudin does not result in a negative Ca^2+^ balance or increased calciotropic hormone levels, suggesting the existence of additional transport pathways or parallel roles for the two claudins. To test this, we generated a *Cldn2/12* double knockout mouse (DKO). These animals have reduced intestinal Ca^2+^ absorption. Colonic Ca^2+^ permeability is also reduced in DKO mice and significantly lower than single-null animals, while small intestine Ca^2+^ permeability is unaltered. The DKO mice display significantly greater urinary Ca^2+^ wasting than *Cldn2* null animals. These perturbations lead to hypocalcemia and reduced bone mineral density, which was not observed in single-KO animals. Both claudins were localized to colonic epithelial crypts and renal proximal tubule cells, but they do not physically interact in vitro. Overexpression of either claudin increased Ca^2+^ permeability in cell models with endogenous expression of the other claudin. We find claudin-2 and claudin-12 form partially redundant, independent Ca^2+^ permeable pores in renal and colonic epithelia that enable paracellular Ca^2+^ (re)absorption in these segments, with either one sufficient to maintain Ca^2+^ balance.

## Significance

Significant calcium absorption across renal and intestinal epithelia occurs via the paracellular pathway. However, the identity of the paracellular pore involved is unknown. Claudin-2 and claudin-12 contribute paracellular calcium permeability in cell models, but single knockout animals don’t have altered serum calcium or bone mineralization. To investigate this, *Cldn2/12* double knockout mice were generated. They display decreased intestinal calcium absorption and renal calcium wasting, resulting in hypocalcemia and markedly reduced bone mineralization. Claudin-2 and claudin-12 don’t physically interact in vitro, and coexpression has an additive effect on calcium permeability. Our work identifies claudin-2 and claudin-12 as important constituents of the paracellular Ca^2+^ pathway in intestine and kidney enabling calcium transport and highlights their important complementary roles in maintaining calcium homeostasis.

Calcium (Ca^2+^) is an essential mineral for physiological processes, including cell signaling, muscle contraction, and bone mineralization. Serum Ca^2+^ is tightly regulated within a narrow range, and homeostasis maintained through coordinated regulation between the intestines, kidneys, and bones (1). Failure to optimally deposit Ca^2+^ into bone results in an increased risk of osteoporosis (2). This disease is responsible for a fracture every 3 s worldwide, presenting a significant economic and healthcare burden globally (3). Moreover, a failure to reabsorb Ca^2+^ along the renal tubule results in increased urinary Ca^2+^ excretion, the greatest risk for kidney stone formation (4). Therefore, understanding the mechanisms of Ca^2+^ balance will aid in understanding and treating these diseases.

Intestinal Ca^2+^ absorption occurs via transcellular or paracellular pathways. Paracellular diffusion is a bidirectional process whereby Ca^2+^ moves down an electrochemical gradient. It predominates when Ca^2+^ intake is adequate (5–7). Ca^2+^ absorption across the small intestine is thought to occur via the paracellular pathway, with the role of the colon still incompletely elucidated (5, 8). However, several reports suggest the colon is essential to Ca^2+^ homeostasis (8–11).

Paracellular permeation of ions across epithelia requires the formation of a pore. Claudins are membrane proteins with two extracellular loops that interact at the tight junction between cells to form pores for, or barriers to, paracellular movement of solutes by altering the charge and size selectivity characteristics (12). In this manner, claudins confer permeability properties to epithelia. Claudins-2, -12, -16, and -19 contribute to the formation of Ca^2+^-permeable paracellular pores. Both claudin-2 and claudin-12 are expressed throughout intestinal epithelia and contribute to Ca^2+^ permeability in cell culture (13). Claudin-2 (*Cldn2*) knockout (KO) mice have decreased colonic Ca^2+^ permeability but unaltered small intestine permeability. They display decreased fecal Ca^2+^ excretion (i.e., increased intestinal Ca^2+^ absorption) and have unaltered bone mineral content and serum Ca^2+^, relative to wild-type (WT) mice (8, 14). The intestinal phenotype of claudin-12 (*Cldn12*) KO mice has not been fully described, although fecal Ca^2+^ and serum Ca^2+^ levels are unaltered relative to WT mice (15). To date, no other claudins have been implicated in forming Ca^2+^ permeable pores in the intestine.

In the kidney, paracellular Ca^2+^ reabsorption occurs across the renal proximal tubule and thick ascending limb. Claudin-2 and claudin-12 are expressed in the proximal tubule, in which two-thirds of filtered Ca^2+^ is reabsorbed (14, 15). Paracellular Ca^2+^ reabsorption is better characterized in the thick ascending limb, in which claudin-16 and claudin-19 form Ca^2+^ permeable pores and mutations in these genes cause the syndrome familial hypomagnesemia with hypercalciuria and nephrocalcinosis associated with severe renal Ca^2+^ wasting (16, 17). In contrast, activation of the Ca^2+^-sensing receptor by increased plasma Ca^2+^ increases the expression of the pore blocking claudin, claudin-14, which prevents Ca^2+^ reabsorption from this segment (18–20).

*Cldn2* KO but not *Cldn12* KO mice have hypercalciuria, although direct measurement of perfused proximal tubules from *Cldn12* KO mice confirm reduced Ca^2+^ permeability (8, 15). Interestingly, neither *Cldn2* KO nor *Cldn*12 KO animals have increased parathyroid hormone (PTH) or calcitriol [1,25(OH)_2_-vitamin D] levels (8, 15). Given that paracellular reabsorption in the proximal tubule is a major contributor to renal Ca^2+^ reabsorption, it is unclear why these phenotypes are not more severe. These data suggest that other yet-to-be-identified pathways exist or that claudin-2 and claudin-12 compensate for each other.

We hypothesized that claudin-2 and claudin-12 form independent cation-permeable pores across intestinal and renal epithelia, contributing independently to paracellular Ca^2+^ transport and maintenance of Ca^2+^ homeostasis. To test this hypothesis, we generated *Cldn2* and *Cldn12* double KO (DKO) mice. Unlike previous models, DKO mice were unable to maintain normal serum Ca^2+^. Decreased Ca^2+^ permeability across the colon but not small intestine resulted in reduced intestinal Ca^2+^ absorption in DKO mice, which also had severe hypercalciuria and markedly decreased bone mineral density. We found that while claudin-2 and -12 were expressed in the same cells, they did not physically interact and coexpression of each claudin had an additive effect on Ca^2+^ permeability in vitro. These results suggest that claudin-2 and -12 mediate paracellular Ca^2+^ absorption and reabsorption independently and that only one of these claudins is sufficient to maintain a normal Ca^2+^ balance.

## Results

### Claudin-2 and Claudin-12 Contribute Paracellular Ca^2+^ Permeability to the Proximal Colon.

**Fig. 1.**
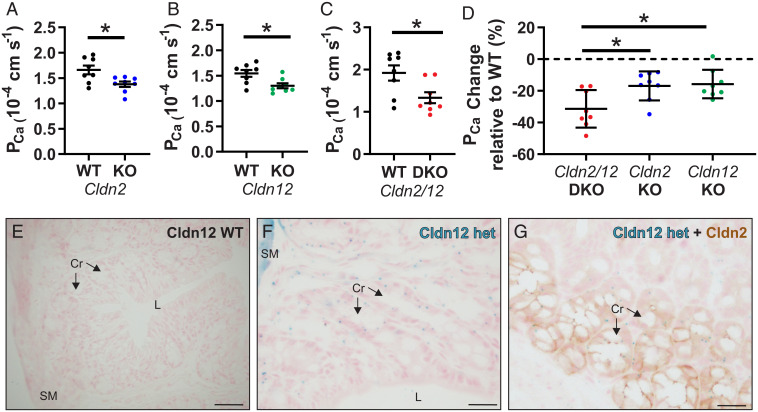
*Cldn2* and *Cldn12* confer independent Ca^2+^ permeability to the proximal colon. P_Ca_ measured ex vivo in Ussing’s chambers across the proximal colon compared to WT of *Cldn2* KO (*A*) (*n* = 8 per group and *P* = 0.016); *Cldn12* KO (*B*) (*n* = 8 per group and *P* = 0.012); and *Cldn2/12* DKO (*C*) (*n* = 8 per group and *P* = 0.019). Full results of bionic dilution potential experiments on small intestine and colon of DKO mice are in *SI Appendix*, Fig. S2 and Tables S1–S4. Means were compared by Student’s *t* test. (*D*) Data from *A–C* expressed as the percentage change in P_Ca_ relative to WT for each genotype. Data are presented as mean ± SD. One-way ANOVA with Dunnett correction for multiple comparisons to compare mean from DKO mice to *Cldn2* KO (*P* = 0.017) and *Cldn12* KO (*P* = 0.010) was performed. The *Cldn12* coding exon was replaced with a LacZ cassette. (*E* and *F*) We used this to localize *Cldn12* expression by X-gal staining of colon from *Cldn12* WT (*E*) and *Cldn12* heterozygous mice (*F*). Staining is present in colonic crypt epithelial cells in *Cldn12* heterozygous mouse (cyan). (Scale bars, 100 µm [*E*] and 25 µm [*F*]). (*G*) X-gal staining of colon for *Cldn12* (cyan) and immunohistochemical staining for claudin-2 (brown) from a *Cldn12* heterozygous mouse. Cr = crypts, SM = smooth muscle, and L = colonic lumen. (Scale bar, 25 µm.) *P < 0.05.

We generated a global *Cldn2* and *Cldn12* DKO mouse by crossing mice from *Cldn2* KO and *Cldn12* KO colonies. DKO mice were born to typically sized litters, and the only observed difference to WT mice was an 8% lower body weight in DKO males (*SI Appendix*, Table S1). We previously reported decreased colonic Ca^2+^ permeability in *Cldn2* KO mice (8). We confirmed this and also found decreased colonic Ca^2+^ permeability in *Cldn12* KO mice (Fig. 1 *A* and *B* and *SI Appendix*, Tables S2 and S3). These results are consistent with both claudin-2 and claudin-12 conferring Ca^2+^ permeability to the proximal colon. We repeated the experiment and found decreased Ca^2+^ permeability across the colon of DKO mice (Fig. 1*C* and *SI Appendix*, Table S4). To determine whether the loss of both *Cldn2* and *Cldn12* in the DKO mice results in an additive loss of Ca^2+^ permeability, we analyzed the results of each KO or DKO relative to their respective WT mice. Colonic Ca^2+^ permeability was decreased by 16.9% (±9.1%) in *Cldn2* KO animals, 16.7% (±9.0%) in *Cldn12* KO animals, and 31.4% (±11.8%) in DKO mice (Fig. 1*D*). The additive results are consistent with claudin-2 and claudin-12 contributing independently to Ca^2+^ permeability across the colon.

The *Cldn12* KO mouse was created by replacing the *Cldn12* coding exon with β-galactosidase (15). Given the lack of sufficient antibodies against claudin-12 (15, 21), we performed X-gal staining on fixed tissue sections to determine the expression of claudin-12 in the mouse colon. X-gal staining (cyan in the section) was present in the crypts of *Cldn12* heterozygous mice but not WT mice (Fig. 1 *E* and *F*). Costaining for claudin-2 (brown) and X-gal (cyan) revealed that both proteins are present but restricted to crypt epithelium (Fig. 1*G* and *SI Appendix*, Fig. S1).

The paracellular pathway is proposed to contribute significant Ca^2+^ absorption from the small intestine. However, prior work found no difference in Ca^2+^ permeability between *Cldn2* KO and WT mice across the small intestine (8). Similarly, in DKO mice, we failed to detect a difference in Ca^2+^ permeability from any small intestinal segment (*SI Appendix*, Fig. S2).

### Claudin-2 and Claudin-12 DKO Mice Exhibit Hypocalcemia, Hypercalciuria, and Decreased Ca^2+^ Balance.

**Fig. 2.**
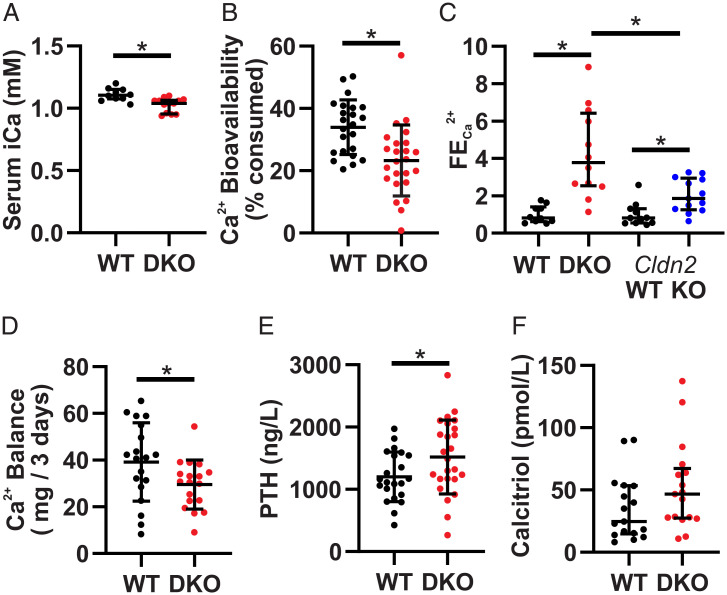
*Cldn2*/*12* DKO mice have hypocalcemia, hypercalciuria, and decreased intestinal Ca^2+^ absorption. (*A*) Serum-ionized Ca^2+^ (median ± interquartile range [IQR], *n* = 10 WT, 13 DKO, Mann–Whitney *U* Test, and *P* = 0.002). (*B*) Ca^2+^ bioavailability as a percent of Ca^2+^ consumed (mean ± SD, *n* = 23 WT, 24 DKO, Student’s *t* test, and *P* = 0.001). (*C*) Fractional excretion of urinary Ca^2+^ normalized to WT for each genotype (median ± IQR, *n* = 10 WT, 12 DKO, 11 *Cldn2* WT, 13 *Cldn2* KO, Mann–Whitney *U* test, *P* < 0.0001 WT versus DKO, *P* = 0.003 *Cldn2* WT versus KO, and *P* = 0.011 DKO versus *Cldn2* KO). (*D*) Net 3 d Ca^2+^ balance (mean ± SD, *n* = 19 WT, 18 DKO, Student’s *t* test, and *P* = 0.043). (*E*) Serum PTH levels (mean ± SD, *n* = 22 WT, 25 DKO, Student’s *t* test, and *P* = 0.039). (*F*) Serum calcitriol (median ± IQR, *n* = 17 WT, 17 DKO, Mann–Whitney *U* Test, and *P* = 0.099). *P < 0.05.

*Cldn2* KO mice exhibit hypercalciuria and increased net intestinal Ca^2+^ absorption, while *Cldn12* KO mice do not have an overt Ca^2+^ phenotype. To ascertain whether DKO mice have a phenotype with altered Ca^2+^ homeostasis, we performed metabolic cage studies. Water and chow consumption, urine volume, and fecal mass were not different between genotypes (*SI Appendix*, Table S1). Blood analysis revealed significantly decreased ionized calcium (iCa) in DKO animals (Fig. 2*A*). No differences in other chemistry, hematology, or blood gas parameters were noted, except that the DKO mice had slightly increased blood sodium, which is unlikely to be physiologically relevant. (*SI Appendix*, Table S5). These results suggest that the DKO mice have a phenotype with altered Ca^2+^ balance.

We measured fecal Ca^2+^ excretion to assess intestinal absorption as a percentage of Ca^2+^ consumed (i.e., bioavailability). DKO mice had decreased Ca^2+^ bioavailability (Fig. 2*B*). This suggests decreased paracellular absorption from the colon, the only intestinal segment with altered Ca^2+^ permeability (Fig. 1 and *SI Appendix*, Fig. S2). Claudin-2 and claudin-12 are also expressed in the renal proximal tubule (*SI Appendix*, Fig. S3). No differences were observed in urinary Na^+^, K^+^, PO_4_^3−^, Cl^−^, or Mg^2+^ excretion. However, we found an almost fourfold increase in Ca^2+^ excretion in the DKO compared to WT mice (*SI Appendix*, Table S5). To account for glomerular filtration and decreased plasma Ca^2+^, we calculated fractional excretion of Ca^2+^ (FECa). Relative to WT mice, the FECa in DKO mice was 4.3-fold greater (Fig. 2*C*). *Cldn2* KO but not *Cldn12* KO mice display hypercalciuria (14, 15). Of note, when normalized to their respective WT mice, FECa of DKO mice was significantly greater than single-*Cldn2* KO animals (Fig. 2*C*). Together, these parameters contribute to decreased overall Ca^2+^ balance in the DKO mice (Fig. 2*D*).

Reduced serum ionized Ca^2+^ in DKO mice should increase serum PTH and, consequently, calcitriol levels. As expected, DKO had increased serum PTH. Despite this, DKO mice did not have a statistically significant increase in serum calcitriol levels (Fig. 2 *E* and *F*). This suggests that DKO mice are unable to appropriately compensate for a decreased Ca^2+^ balance.

### Renal and Intestinal Gene Expression Changes in DKO Mice.

We examined expression of renal genes involved in Ca^2+^ reabsorption to assess whether compensation for marked hypercalciuria occurs. Expression of *Cldn14* and *Cldn16,* which respectively blocks and permits Ca^2+^ reabsorption in the thick ascending limb, were slightly increased (*SI Appendix*, Fig. S4 *A–C*) (18, 22). Expression of *Trpv5*, *Calb1*, and *Slc8a1*, which contribute to transcellular reabsorption from the distal nephron, was also increased in DKO mice; there was also an increased abundance of intracellular binding protein calbindin-D_28k_ (encoded by *Calb1*; *SI Appendix*, Fig. S4 *D–I*). *Cyp27b1* and *Cyp24a1*, genes encoding enzymes that activate and deactivate calcitriol, respectively, were not significantly increased in DKO mice (*SI Appendix*, Fig. S4 *J* and *K*).

In the colon, although the expression of the apical Ca^2+^ channels *Trpv6* and *Cacna1d* were not changed, the expression of the intracellular Ca^2+^-binding protein *S100g* and basolateral extrusion pump *Atp2b1* were increased by fivefold and 0.5-fold, respectively. We observed no change in *Cldn3* or *Cldn4* expression but found a slight decrease in the expression of *Cldn15*, a gene that encodes a cation-permeable pore (23). No changes were observed in the duodenum (*SI Appendix*, Fig. S5). These results altogether suggest that the DKO mice have increased transcellular Ca^2+^ absorption from the colon, which is not stimulated by calcitriol. The intestinal expression of genes encoding Ca^2+^ transporters have not been reported for the *Cldn12* KO mouse. We therefore measured them and found that the only difference was a twofold increase in *S100g* expression in the colon (*SI Appendix*, Fig. S6). Taken together, the results indicate that the DKO mice have impaired intestinal Ca^2+^ absorption and renal reabsorption with insufficient compensation to maintain serum iCa.

### Claudin-2 and Claudin-12 DKO Mice Have Reduced Bone Mineralization.

**Fig. 3.**
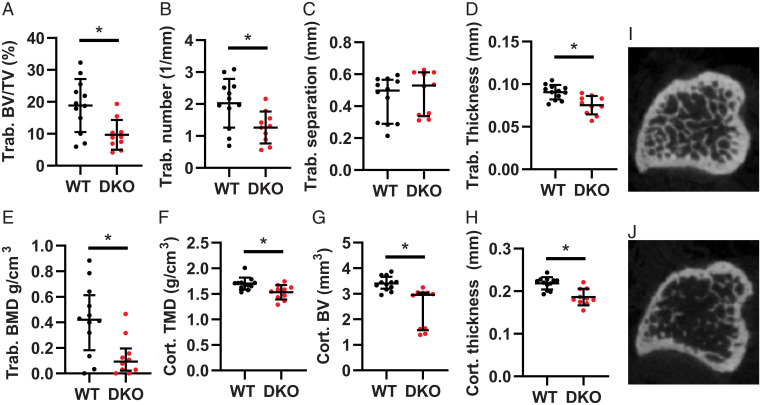
*Cldn2/12* DKO mice have altered bone morphometry at 3 mo. Microarchitecture of trabecular (trab) (*A–E*) and cortical (cort) (*F*–*H*) bone from tibia of WT and *Cldn2/12* DKO mice analyzed by micro-CT. (*A*) Trabecular bone volume/tissue volume (*P* = 0.006). (*B*) Trabecular number (*P* = 0.014). (*C*) Trabecular separation (*P* = 0.228). (*D*) Trabecular thickness (*P* = 0.001). (*E*) Trabecular bone mineral density (*P* = 0.014). (*F*) Cortical tissue mineral density (*P* = 0.006). (*G*) Cortical bone volume (*P* = 0.0001). (*H*) Cortical thickness (*P* = 0.0003). Representative micro-CT images of the tibial metaphyses shown at 40 slices from growth plate from WT (*I*) and DKO (*J*) mice. Data are presented as mean ± SD compared by unpaired *t* test (*A*, *B*, *D*, *G*, and *H*) or as median (IQR) compared by Mann–Whitney *U* test (*C*, *E*, and *F*). *P < 0.05.

Bone mineralization and microarchitecture is not altered in *Cldn2* KO mice but has not been examined in *Cldn12* KO mice (8). We therefore performed microcomputed tomography (µCT) studies on *Cldn12* KO mice at 3 and 6 mo of age and observed no differences to WT (*SI Appendix*, Table S6 and S7). We next examined bone microarchitecture and mineral density in the DKO mice and found a more-than-fourfold decrease in trabecular bone mineral density and both decreased trabecular thickness and number in DKO mice. Similarly, we found decreased bone volume, thickness, and tissue mineral density in cortical bone of DKO mice (Fig. 3). These results are consistent with reduced bone mineralization or accelerated demineralization in DKO mice and support the notion that claudin-2 and -12 compensate for one another when one is lost.

### Claudin-2 and -12 Do Not Physically Interact In Vitro yet Confer Increased Ca^2+^ Permeability.

**Fig. 4.**
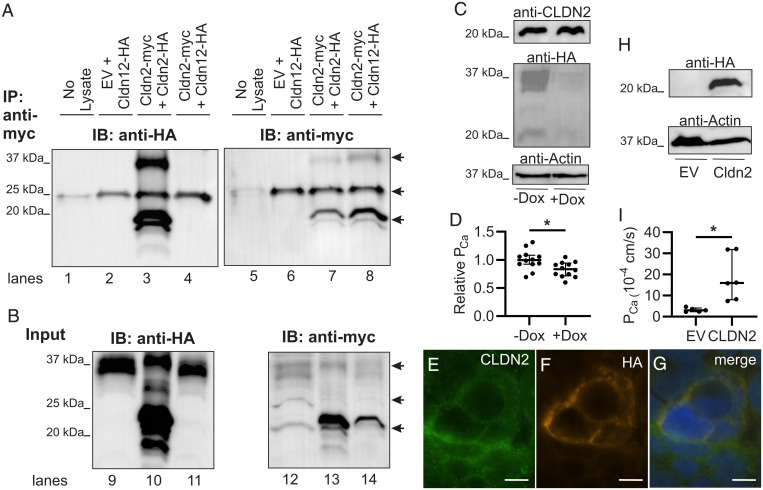
Claudin-2 and claudin-12 form separate Ca^2+^ permeable pores. (*A*) HEK293 cells transfected as indicated were lysed or the buffer without cell lysate as control (no lysate lane) and proteins immunoprecipitated (IP) with antibodies to myc before sodium dodecyl sulphate–polyacrylamide gel electrophoresis (SDS-PAGE). Proteins were electrotransferred and blotted with anti-HA antibody (lanes 1 through 4) and then stripped and reprobed with an anti-myc antibody (lanes 5 through 8). IB, immunoblot. (*B*) Protein from HEK293 cells above directly resolved on SDS-PAGE (Input) and blotted with anti-HA (lanes 9 through 11) and then anti-myc (lanes 12 through 14). Claudin dimers (37 kDa) and monomers (20 kDa) are highlighted by arrows. The arrow at 25 kDa illustrates IgG light chain in the IP samples as seen in the lane with no cell lysate added. EV, empty vector. Representative blot of three repeats is shown. (*C*) Caco-2 cells endogenously express CLDN2 and express HA-tagged CLDN12 via a tet-off system. (*D*) Caco-2 cells expressing CLDN2 and CLDN12 (−Dox) have greater Ca^2+^ permeability than cells expressing only CLDN2 (+Dox) (*P* = 0.014 and unpaired *t* test). (*E*–*G*) Immunofluorescence of Caco-2 cells for endogenous CLDN2 (green) and HA-tagged CLDN12 (orange) demonstrating colocalization of CLDN2 and CLDN12 in the same cell membrane. (*H*) Opossum kidney (OK) cells endogenously expressing CLDN12 and expressing either EV or CLDN2. (*I*) OK cells expressing CLDN2 and CLDN12 (“CLDN2” data) have greater Ca^2+^ permeability than cells expressing only CLDN12 (“EV” data) (*P* = 0.018, Welch’s *t* test). Dox, doxycycline. TER, pNa, pCl, and relative permeabilities are presented in *SI Appendix*, Tables S8 and S9. *P < 0.05.

Given that the loss of claudin-2 and -12 have an additive effect on Ca^2+^ permeability and urinary Ca^2+^ excretion, we hypothesized that these proteins form independent pores. To assess this, we performed coimmunoprecipitation studies using human embryonic kidney (HEK293) cells expressing epitope-tagged claudin-2 and claudin-12. As a positive control, we found that myc-tagged claudin-2 was able to immunoprecipitate hemagglutinin (HA)-tagged claudin-2. However, myc-tagged claudin-2 was unable to immunoprecipitate HA-tagged claudin-12 (Fig. 4 *A* and *B*). Together, these results are consistent with claudin-2 and claudin-12 forming separate Ca^2+^ permeable pores.

If claudin-2 and claudin-12 form separate Ca^2+^ permeable pores in renal and intestinal epithelia, Ca^2+^ permeability should be greater with both proteins present than with only one. We therefore established a colonic epithelial cell line, Caco-2, that stably expressed HA-tagged CLDN12 under a tet-off system. *CLDN2* is endogenously present in these cells (Fig. 4*C*). After doxycycline treatment, CLDN12 was only very faintly detected (Fig. 4*C*). Colonic epithelial cells expressing both CLDN2 and CLDN12 had higher Ca^2+^ permeability than cells only expressing CLDN2 (Fig. 4*D* and *SI Appendix*, Table S8). Immunofluorescence of this cell line demonstrates colocalization of CLDN2 and CLDN12 at the plasma membrane (Fig. 4 *E–G*). Similarly, we employed a renal proximal tubule cell line (opossum kidney cells) that endogenously expresses CLDN12 but not CLDN2 and expressed either the empty vector or a HA-tagged CLDN2 (Fig. 4*H*) (24). Proximal tubule cells expressing both CLDN2 and CLDN12 had significantly greater Ca^2+^ permeability than cells only expressing CLDN12 (Fig. 4*I* and *SI Appendix*, Table S9). These results are consistent with claudin-2 and claudin-12 forming separate Ca^2+^-permeable pores.

## Discussion

Employing murine models, detailed balance studies, and ex vivo and in vitro techniques, we provide evidence that either claudin-2 or claudin-12 is necessary to maintain Ca^2+^ homeostasis and optimal bone mineralization. This conclusion is supported by *Cldn2/Cldn12* DKO mice having decreased Ca^2+^ permeability across the colon, contributing decreased net intestinal Ca^2+^ absorption. Furthermore, DKO mice have even greater hypercalciuria than the single-*Cldn2* KO animals. Intestinal and renal compensatory mechanisms in the DKO mice are inadequate to maintain serum ionized Ca^2+^ levels. Ultimately, the loss of both proteins results in decreased bone mineral density. Moreover, we did not find that claudin-2 and -12 physically interact, despite being expressed in the same epithelia. Our results are consistent with additive effects of their deletion and their formation of independent Ca^2+^ pores in the proximal colon and renal proximal tubule.

Paracellular intestinal Ca^2+^ absorption is proposed as the predominant pathway when dietary Ca^2+^ is adequate (5). However, this has never been directly tested. Claudin-2 and -12 have been implicated in mediating paracellular Ca^2+^ diffusion between epithelial cells (13, 15, 25). Herein, we describe a genetic model in which these two tight junction proteins are deleted. The DKO mice display decreased intestinal Ca^2+^ absorption when fed an adequate Ca^2+^-containing diet, likely due to reduced colonic Ca^2+^ permeability. Previous work clearly demonstrates Ca^2+^ absorption via the paracellular pathway from the large intestine under normal and high-dietary Ca^2+^ conditions (6, 7, 26). However, this paracellular pathway can mediate bidirectional diffusion of Ca^2+^, and thus, Ca^2+^ secretion has also been observed (26, 27). Therefore, depending on the electrochemical properties across the tissue, net secretion or absorption may occur. Previous work found that the loss of *Cldn2* leads to increased net Ca^2+^ absorption, likely due to reduced colonic Ca^2+^ secretion (8). Despite reduced colonic Ca^2+^ permeability, *Cldn12* KO mice do not have altered net intestinal Ca^2+^ absorption (15). In contrast to the single-KO animals, we observe decreased net Ca^2+^ absorption in DKO mice. Moreover, again in contrast to the single-KO mice, the DKO animals demonstrate reduced plasma Ca^2+^ levels and reduced bone mineral density. In *Cldn2* KO mice, we only identified changes in Ca^2+^ permeability across the colon. However, in DKO and *Cldn12* KO mice, transepithelial resistance was increased, and absolute Na^+^ and Cl^−^ permeabilities as well as relative cation permeability were decreased. These alterations may contribute to an altered electrochemical driving force across the colon and explain the differences observed between *Cldn2* KO and DKO mice. Regardless, our work provides evidence supporting a significant role for paracellular colonic Ca^2+^ absorption in the maintenance of Ca^2+^ homeostasis.

The DKO mice display marked hypercalciuria. Both claudin-2 and claudin-12 contribute Ca^2+^ permeability to the proximal tubule (15, 28). Perfused proximal tubules from *Cldn12* KO mice have decreased Na^+^ and Ca^2+^ permeability but unaffected Cl^−^ permeability (15), whereas both *Cldn2* KO and *Cldn12* KO proximal tubules are anion selective in contrast to proximal tubules from WT mice, which are cation selective (14, 15). However, *Cldn12* KO mice do not have increased urinary Ca^2+^ excretion, perhaps due to compensation (15). Conversely, *Cldn2* KO mice have a threefold increase in fractional Ca^2+^ excretion compared to WT (14, 18). The DKO mice have even greater renal Ca^2+^ wasting. The loss of claudin-2 and claudin-12 likely results in markedly inadequate Ca^2+^ reabsorption from the proximal tubule that overwhelms the compensatory capacity of the more distal segments.

A physical interaction between claudin-16 and -19 is required for the formation of a Mg^2+^- and Ca^2+^-permeable pore in the renal thick ascending limb (22, 29, 30). This physical coupling is highlighted by the fact that the loss of either claudin-16 or claudin-19 results in the same disease, familial hypomagnesemia with hypercalciuria and nephrocalcinosis (16, 17). In contrast, the loss of claudin-2 or another cation-permeable claudin, claudin-15, both result in decreased sodium permeability across the small intestine, and loss of both together appears to have an additive effect (23, 31). Thus, claudin-2 and -15 appear to form independent, cation-permeable pores across intestinal epithelia. Similarly, we have now provided evidence that claudin-2 and claudin-12 form independent, cation-permeable pores across renal and intestinal epithelia.

Previous in vitro work identified two other claudins, claudin-10a and claudin-3, predominantly expressed in the renal proximal tubule. Claudin-2 was found to weakly interact with claudin-10a but not with claudin-3 (28), and the interaction between claudin-2 and -10a is not within the same pore but through their formation of independent, parallel pores in the tight junction (28). Our findings regarding claudin-2 and -12 are consistent with the two claudins analogously being present in the same cell but forming independent pores.

The altered bone microarchitecture and decreased bone mineral density observed in the DKO mice is likely due to both inadequate intestinal absorption and renal reabsorption of Ca^2+^ as the effects of genetic alterations disrupting either intestinal absorption or renal reabsorption can be compensated by the other organ. Delineating the relative contribution of altered intestinal Ca^2+^ absorption or renal reabsorption in the DKO mice is an important future direction. However, this requires the generation of floxed models to allow for Cre-dependent, tissue-specific intestinal or renal DKO mice to tease apart these organ-specific effects. *Cldn2* and *Cldn12* are expressed in osteoblasts, although their function in bone is yet to be elucidated (32, 33). However, if our observed phenotype was due primarily to altered bone homeostasis, it is likely we would have observed greater intestinal Ca^2+^ absorption or renal reabsorption due to compensation rather than the decreased levels we observed. Regardless of the relative contributions of altered renal or intestinal paracellular Ca^2+^ absorption to the observed phenotype, our results support a significant role for the paracellular pathway in intestinal Ca^2+^ absorption under adequate dietary intake.

In summary, we present evidence that the loss of paracellular Ca^2+^ permeability across the colon and proximal tubule leads to a decreased net Ca^2+^ balance, decreased bone mineralization, and inability to maintain serum Ca^2+^ levels. Our work supports two independent but partially redundant, paracellular pathways mediated by claudin-2 and claudin-12. Overall, we highlight the critical role of claudins-2 and -12 paracellular Ca^2+^ pores in the colon and proximal tubule in maintaining Ca^2+^ balance.

## Materials and Methods

The collection of serum, urine, and feces and measurement of electrolytes and calciotropic hormones has been described previously (15). Real-time qPCR, Ca^2+^ permeability of intestinal tissue, microcomputed tomography, immunofluorescence staining, and immunoblotting were performed as previously (8, 15, 18, 34–40). Refer to *SI Appendix*, *Supplementary Methods* for detailed descriptions.

### Ethics Approval.

Experiments were approved by the University of Alberta Research Ethics Board animal ethics committee, Health Sciences Section (AUP00000213).

### Animals and Husbandry.

We generated *Cldn2* and *Cldn12* global DKO mice (*Cldn2/12* DKO) by cross-breeding *Cldn2* (MMRRC, University of California, Davis) with *Cldn12* KO (15) animals which had been backcrossed onto an FVB/N WT background (Taconic Biosciences, Rensselaer). DKO genotyping was confirmed by real-time qPCR as described in *SI Appendix*, *Supplementary Methods* using kidney and intestinal tissue. *Cldn2* and *Cldn12* gene expression was detected in WT but not DKO animals, whereas β-galactosidase was detected in tissue of DKO animals only. N.B. *Cldn12* KO animals were generated by homologous recombination of exon 4 of the *Cldn12* gene with the β-galactosidase coding sequence from *Escherichia coli*, and therefore, this gene will only be detected in mice carrying the mutant form (15). Metabolic cage studies were performed as previously described (18, 34).

### Coimmunoprecipitation.

HEK293 cells (ATCC) were transiently transfected with pTRE2hyg vector expressing HA-tagged claudin-12, pTRE2hyg vector expressing myc-tagged claudin-2, pcDNA 3.1 vector expressing HA-tagged claudin-2, and/or empty pTRE2hyg vector as described in the Fig. 4 legend. Cells were lysed in a EDTA and nonidet P-40 containing Tris buffer, and lysate was incubated with mouse anti-myc antibody (Thermo Fisher Scientific, catalog No. MAI-21316) and precipitated with Dynabeads with protein G (magnetic beads, Invitrogen) overnight at 4 °C. Protein was eluted with Laemmli buffer and detected by immunoblot as above with rat anti-HA (Roche, catalog No. 11867423001) or rabbit anti-myc (Covance catalog No. PRB-150P) antibodies.

### Immunohistochemistry and X-gal Staining.

Tissue was subjected to X-gal staining and, subsequently, immunohistochemistry, as described in detail previously (19). Briefly, sections of colon and kidney tissue were fixed in 4% PFA, cryoprotected in sucrose, and frozen before being sectioned and stained for X-gal. Some of the X-gal–stained sections were subsequently stained for CLDN2 using rabbit anti-CLDN2 antibodies (#51–6100, Invitrogen).

### Statistics.

Data were analyzed using GraphPad Prism 9.0. The Shapiro–Wilk test was used to evaluate for normal distribution and F test to compare variances. Data were analyzed and presented as indicated in figure and table legends. *P* < 0.05 was considered statistically significant.

## Supplementary Material

Supplementary File

## Data Availability

All study data are included in the article and/or *SI Appendix*.
