# Supplementary material for: Claudin-2 and claudin-12 form independent, complementary pores required to maintain calcium homeostasis
Source: Proc Natl Acad Sci U S A. 2021 Nov 22;118(48):e2111247118. doi: 10.1073/pnas.2111247118 (PMC8694054; doi:10.1073/pnas.2111247118)
Supplement: Supplementary File [file pnas.2111247118.sapp.pdf]

Supplemental Information Appendix

Table S1. Metabolic cage data

	**Male**			**Female**		
	**WT**	**DKO**	***P*- value**	**WT**	**DKO**	***P*- value**
N	15	19		19	18	
Body Weight (g)	31.3 ± 4.52	28.5 ± 2.73	0.0346	25.4 ± 3.48	24.3 ± 2.88	0.2925
Chow eaten (g/g body weight)	0.12 ± 0.05	0.14 ± 0.05	0.2146	0.17 ± 0.05	0.17 ± 0.05	0.8337
Water intake (mL/24 hour/g body weight)	0.16 ± 0.08	0.15 ± 0.04	0.8252	0.15 ± 0.04	0.16 ± 0.04	0.2801
Urine volume (mL/24 hour/g body weight)	0.074 ± 0.047	0.080 ± 0.026	0.6673	0.043 ± 0.016	0.060 ± 0.039	0.0878
Fecal excretion, wet (g/24 hour/g body weight)	0.012 ± 0.004	0.015 ± 0.006	0.2459	0.017 ± 0.005	0.018 ± 0.005	0.5585

Data presented as mean ± SD compared by unpaired t-test.

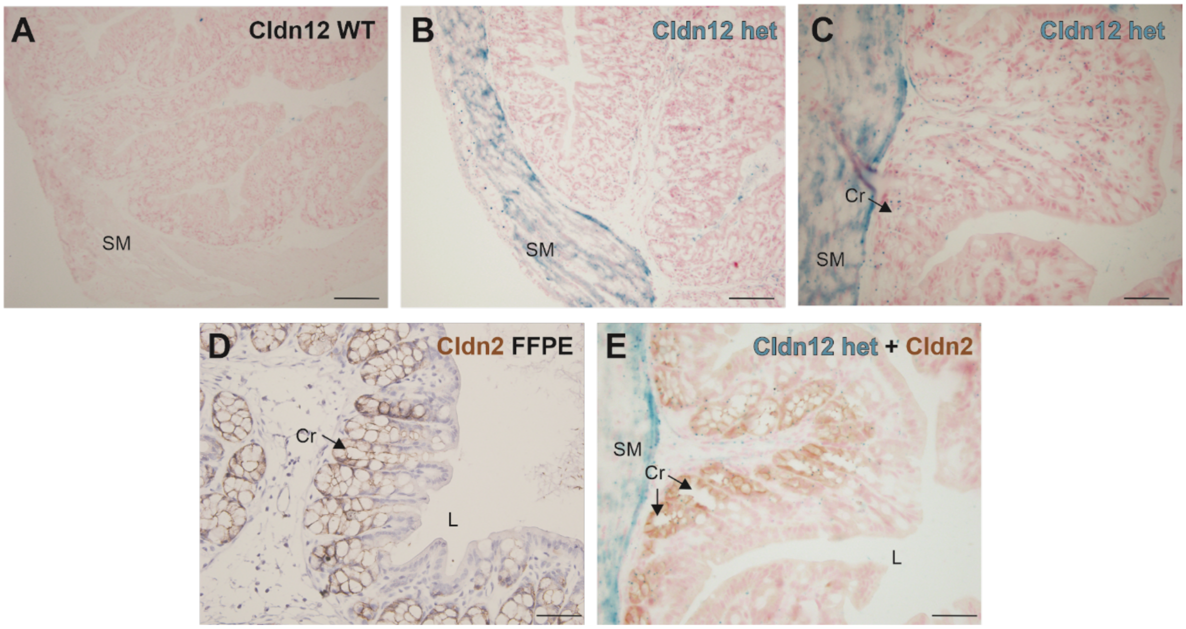

Figure S1. Claudin-2 and claudin-12 are expressed together in colonic crypt epithelial cells. A-C) X-gal staining of fixed and frozen colon sections from WT (A) and *Cldn12* heterozygous mice (B-C). X-gal staining originates from a LacZ cassette used to ablate the *Cldn12* gene and stains cyan. D) CLDN2 (brown) staining of formalin-fixed paraffin embedded colon section from a wild-type mouse. E) Co-staining with X-gal (cyan) and CLDN2 (brown) in *Cldn12* heterozygous mice. Cr = crypts, SM = smooth muscle, L = colonic lumen. Scale bars 200 µm (A, B) and 50 µm (C-E).

Table S2. Proximal colon resistance and ion permeability of WT and *Cldn2* KO mice

	**WT** (n = 8)	***Cldn2* KO** (n = 8)	***P* – value**
**TER (Ω cm^2)**	55.4 ± 7.20	59.5 ± 5.83	0.2377
**P_{Na} (x10^{-4} cm s^{-1})**	0.261 ± 0.031	0.243 ± 0.027	0.2581
**P_{Cl} (x10^{-4} cm s^{-1})**	0.232 ± 0.037	0.212 ± 0.022	0.2143
**P_{Na}/P_{Cl}**	1.14 ± 0.11	1.16 ± 0.13	0.7605
**P_{Ca}/P_{Na}**	6.36 ± 0.403	5.68 ± 0.353	0.0029

TER, transepithelial resistance; P_x, permeability to ion x. Data presented as mean ± SD. *P*–value determined by unpaired t-test.

Table S3. Proximal colon resistance and ion permeability of WT and *Cldn12* KO mice

	**WT** (n = 8)	***Cldn12* KO** (n = 8)	***P* – value**
**TER (Ω cm^2)**	52.4 ± 5.29	62.1 ± 5.84	0.0035
**P_{Na} (x10^{-4} cm s^{-1})**	0.267 ± 0.023	0.232 ± 0.022	0.0075
**P_{Cl} (x10^{-4} cm s^{-1})**	0.253 ± 0.033	0.203 ± 0.024	0.0040
**P_{Na}/P_{Cl}**	1.07 ± 0.07	1.14 ± 0.06	0.0308
**P_{Ca}/P_{Na}**	5.77 ± 0.417	5.65 ± 0.474	0.6053

TER, transepithelial resistance; P_x, permeability to ion x. Data presented as mean ± SD. *P*–value determined by unpaired t-test.

Table S4. Proximal colon resistance and ion permeability of WT and *Cldn2/12* DKO mice

	WT (n = 8)	*Cldn2/12* DKO (n = 8)	***P* – value**
**TER (Ω cm^2)**	44.0 ± 12.3	61.1 ± 17.4	0.0392
**P_{Na} (x10^{-4} cm s^{-1})**	0.307 ± 0.055	0.249 ± 0.049	0.0427
**P_{Cl} (x10^{-4} cm s^{-1})**	0.338 ± 0.082	0.239 ± 0.065	0.0182
**P_{Na}/P_{Cl}**	0.923 ± 0.071	1.06 ± 0.150	0.0330
**P_{Ca}/P_{Na}**	6.17 ± 0.676	5.37 ± 0.864	0.0692

TER, transepithelial resistance; P_x, permeability to ion x. Data presented as mean ± SD. *P* – value determined by unpaired t-test.

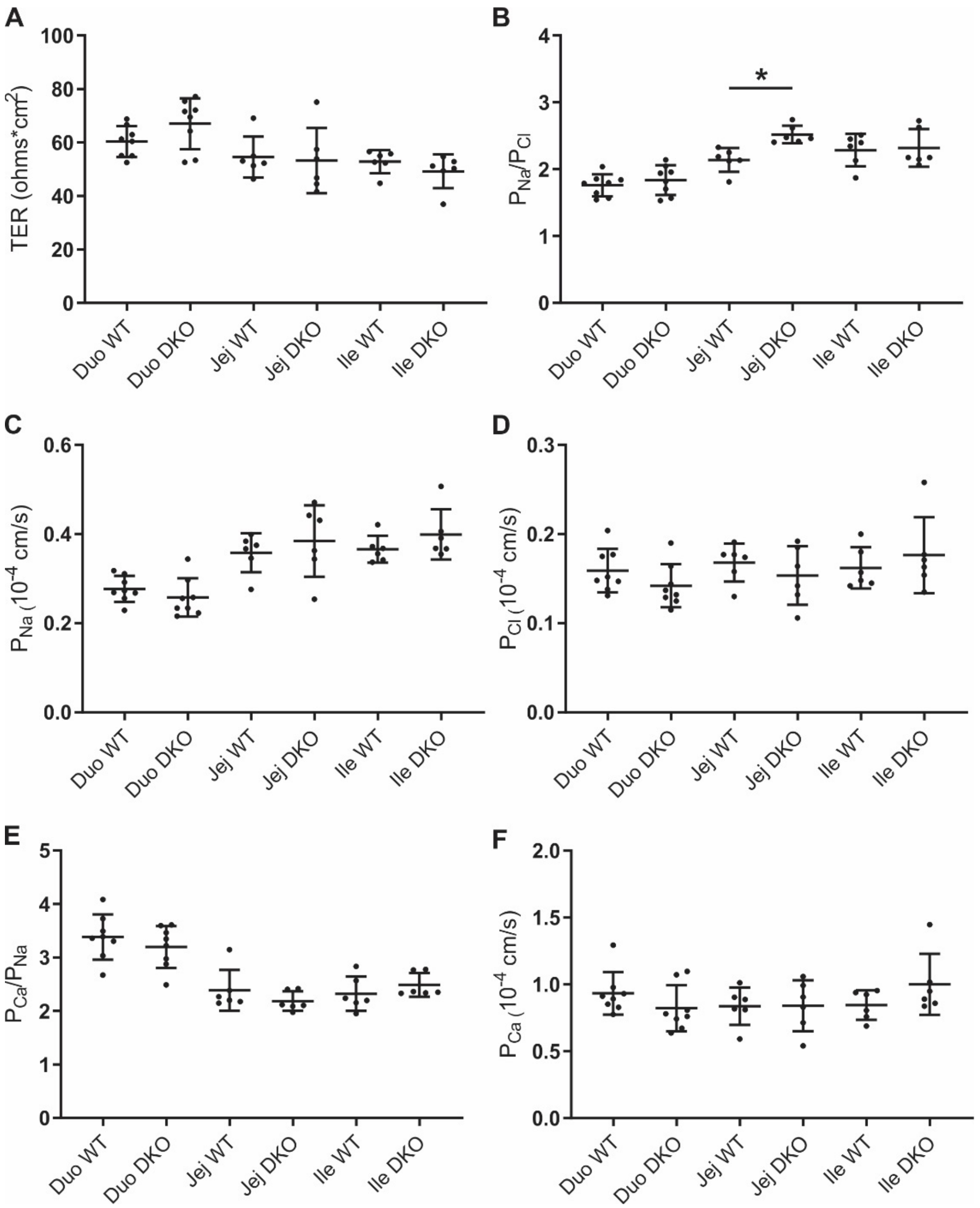

Figure S2. Calcium permeability is not different from wild-type animals along the small intestine of *Cldn2/12* DKO mice. A) Transepithelial resistance, B) and E) permeability ratios, C, D, F) absolute ion permeability of *Cldn2/12* DKO mice vs WT for each intestinal segment. Duo, duodenum; Jej, jejunum; Ile, ileum. N = 8 per group for Duo and 6 per group for Jej and Ile. Data presented as mean ± SD, comparisons by unpaired t-test * $P < 0.05$.

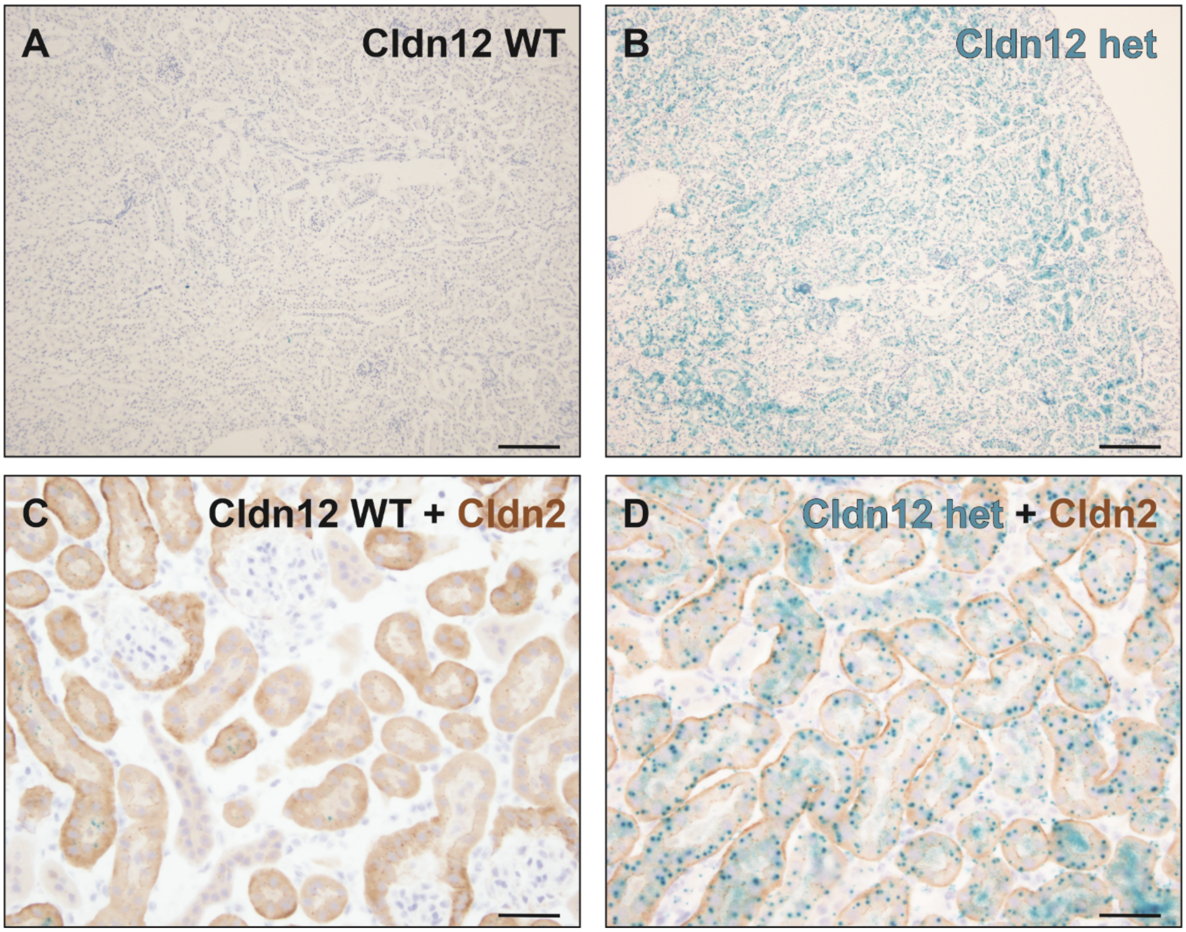

Figure S3. Claudin-2 and claudin-12 are expressed together in renal proximal tubules. X-gal staining of frozen kidney sections from A) WT and B) *Cldn12* heterozygous mice. Nuclei are counterstained with hematoxylin (blue). X-gal staining represents claudin-12 (cyan) expression in heterozygous mice, which is absent in WT mice. C-D) Co-staining with X-gal (cyan) and CLDN2 (brown) in WT (C) and *Cldn12* heterozygous mice (D). Scale bars 200 nm (A, B), and 50 nm (C, D).

Table S5. Serum and urine biochemistries

	N	WT	N	*Cldn2/12* DKO	*P*-value
**Serum**					
Sodium (mM)	34	146 (144 – 148)	37	147 (146 – 149)	0.0201
Potassium (mM)	34	4.8 (4.5 – 5.2)	37	4.7 (4.3 – 5.1)	0.2689
Chloride (mM)	34	116 (113.8 – 119)	37	116 (114 – 119.5)	0.7411
TCO_2 (mM)	34	22.7 ± 3.1	37	22.8 ± 2.4	0.8766
BUN (mM)	34	24.6 ± 5.9	37	27.4 ± 6.2	0.0543
Glucose (mM)	34	10.2 ± 2.2	37	10.0 ± 1.7	0.5846
Hct (%PCV)	34	41.0 (39.0 – 42.0)	37	41.0 (39.5 – 43.0)	0.2146
Hgb (g/L)	34	139 (133 – 143)	37	139 (134 – 146)	0.2146
pH	24	7.28 ± 0.05	24	7.30 ± 0.03	0.0509
pCO_2 (mmHg)	24	47.0 ± 5.6	24	45.6 ± 3.4	0.3045
HCO_3 (mM)	24	22.0 ± 2.7	24	22.6 ± 1.8	0.4982
BE (mM)	24	-5 ± 3	24	-4 ± 2	0.2148
Anion Gap (mM)	34	13.3 ± 3.0	37	13.9 ± 2.4	0.3190
Creatinine (µM)	10	18.2 ± 6.3	12	19.3 ± 6.8	0.6824
**Urine**					
Ca^{2+} (/Creatinine)	24	0.91 (0.50 – 1.63)	24	2.67 (1.65 – 4.23)	< 0.0001
Cl^- (/Creatinine)	24	102 (80 – 133)	24	121 (82 – 141)	0.4284
PO_4^{3-} (/Creatinine)	24	106 (85 – 149)	24	131 (98 – 156)	0.2912
Mg^{2+} (/Creatinine)	24	3.8 (2.8 – 5.6)	24	4.4 (3.6 – 5.5)	0.3152
K^+ (/Creatinine)	24	179 (156 – 230)	24	223 (171 – 271)	0.1065
Na^+ (/Creatinine)	24	131 (109 – 151)	24	152 (111-192)	0.3203
Ca^{2+} (mg in 24 hours)	24	0.12 (0.07 - 0.21)	24	0.41 (0.25 – 0.50)	<0.0001
Cl^- (mg in 24 hours)	24	13.9 (9.2 – 19.0)	24	15.2 (11.2 – 19.8)	0.7247
PO_4^{3-} (mg in 24 hours)	24	34.3 (29.2 – 55.2)	24	47.5 (33.5 – 57.2)	0.2143
Mg^{2+} (mg in 24 hours)	24	0.35 (0.25 – 0.49)	24	0.43 (0.30 – 0.50)	0.3305
K^+ (mg in 24 hours)	24	28.5 (20.7 – 35.7)	24	31.7 (26.4 – 44.6)	0.2465
Na^+ (mg in 24 hours)	24	11.9 (7.5 – 14.5)	24	13.2 (10.1 – 17.7)	0.4677

Data presented as mean ± SD compared by unpaired t-test, or as median (IQR) compared by Mann-Whitney test. TCO_2, total carbon dioxide; BUN, blood urea nitrogen; Hct, hematocrit; Hgb, hemoglobin; pCO_2, partial pressure of carbon dioxide; HCO_3, bicarbonate; BE, base excess.

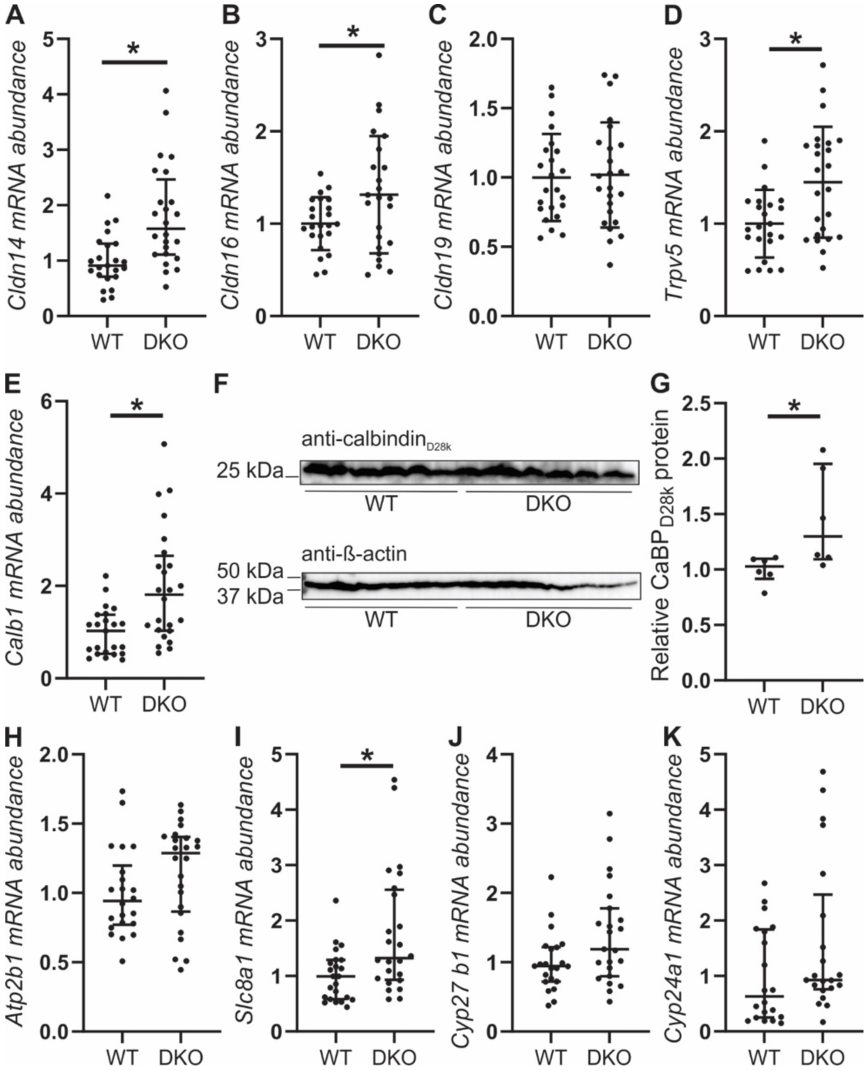

Figure S4. ***Cldn2/12*** **DKO renal gene expression suggests compensatory increased Ca^{2+} reabsorption from the distal nephron.** mRNA abundance of renal genes expressing A) claudin-14 ($P = 0.0003$), B) claudin-16 ($P = 0.0354$), C) claudin-19 ($P = 0.8567$), D) TRPV5 ($P = 0.0035$), E) calbindin-D_{28k} ($P = 0.0016$). F) Representative immunoblots from kidney of WT and DKO mice probed with anti-calbindin-D_{28k} and β-actin and G) semi-quantification of the immunoblot ($P = 0.0152$, N = 6 each group). mRNA abundance of H) PMCA1b ($P = 0.1218$), I) NCX1 ($P = 0.0051$), J) *Cyp27b1* encoding 1α-hydroxylase ($P = 0.0516$), and K) *Cyp24a1* encoding 24-hydroxylase ($P = 0.0782$) in the kidney of WT and *Cldn2/12* DKO mice. Results are normalized to *β-Actin* and expressed relative to WT. Data presented as median ± IQR and compared using Mann-Whitney test (A, E – I), data presented as mean ± SD and compared by Welch's t-test (B – D). n = 23 WT and 24 DKO.

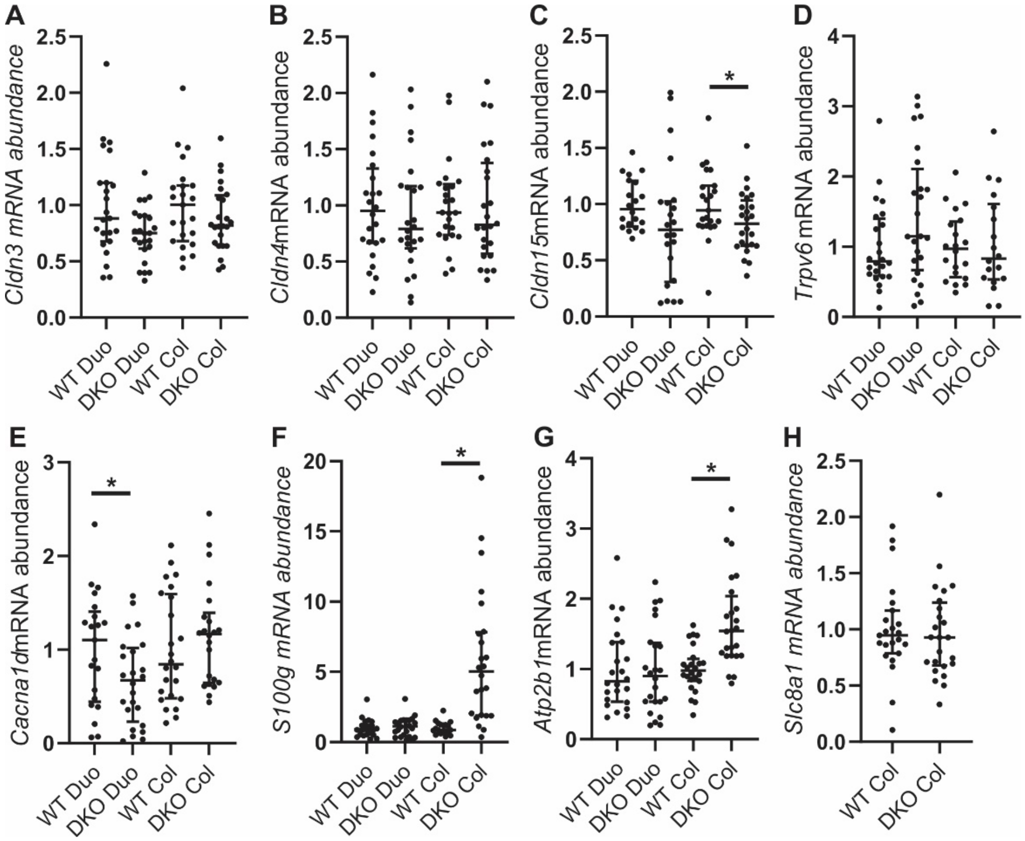

Figure S5. *Cldn2/12* DKO intestinal gene expression suggests compensatory increases in transcellular Ca^{2+} from the proximal colon. mRNA abundance of genes expressing A) claudin-3, B) claudin-4, C) claudin-15, D) *Trpv6*, E) $Ca_v1.3$, F) calbindin-D_{9k}, G) Pmca1b, H) Ncx1 in the intestine of WT and *Cldn2/12* DKO mice. Expression normalized to *β-actin* and expressed relative to WT for each intestinal segment. Duo, duodenum; Col, colon. n = 23 WT and 24 DKO. Data presented as median ± IQR. Comparison of DKO to WT for each intestinal segment by Mann-Whitney test. *$P < 0.05$

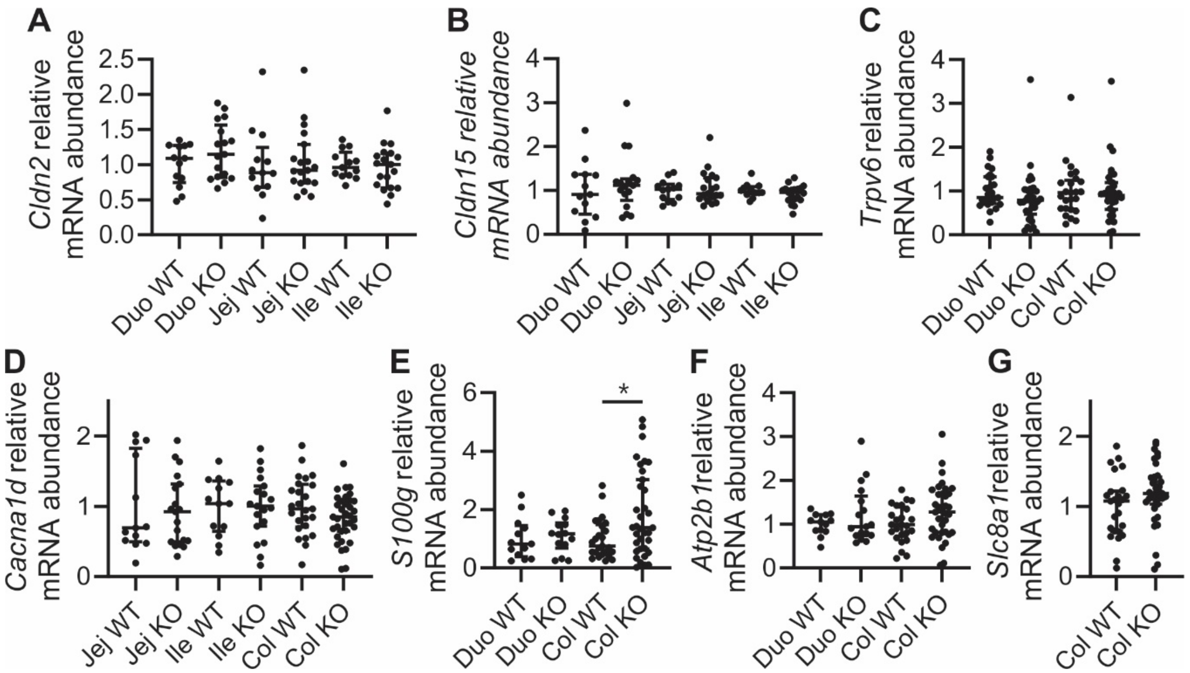

Figure S6. Gene expression in *Cldn12* KO intestine. mRNA abundance of genes expressing A) claudin-2, B) claudin-15, C) TRPV6, D) Ca_v1.3, E) calbindin-D_{9k}, F) PMCA1b, G) NCX1 in the intestine of WT and *Cldn12* KO mice. Expression normalized to *Gapdh* (Duo, Jej, Ile) or *18S* (Col) and expressed relative to WT for each intestinal segment. Duo, duodenum; Jej, jejunum; Ile, ileum; Col, colon. n = 13 - 35. Data presented as median ± IQR. Comparison of KO to WT for each intestinal segment by Mann-Whitney test. $^*P < 0.05$

Table S6. Trabecular and cortical bone parameters of 3-month-old *Cldn12* KO vs. WT mice

	Male			Female		
	**WT**	***Cldn12* KO**	**P- value**	**WT**	***Cldn12* KO**	**P- value**
N	8	7		7	9	
**Trabecular Bone**						
Bone Mineral Density (g/cm^3)	0.193 ± 0.041	0.223 ± 0.069	0.3076	0.200 ± 0.024	0.197 ± 0.060	0.9027
BV/TV (%)	11.38 ± 4.20	17.10 ± 9.55	0.1805	11.13 ± 3.00	11.97 ± 8.56	0.7891
Trabecular Thickness (mm)	0.066 ± 0.006	0.075 ± 0.012	0.0769	0.079 ± 0.006	0.078 ± 0.020	0.9242
Trabecular Separation (mm)	0.403 ± 0.124	0.331 ± 0.159	0.3385	0.520 ± 0.126	0.457 ± 0.136	0.5197
Trabecular Number (1/mm)	1.68 ± 0.539	2.16 ± 1.04	0.2745	1.40 ± 0.344	1.48 ± 0.852	0.7943
**Cortical Bone**						
Bone Volume (mm^3)	1.87 ± 0.210	1.96 ± 0.262	0.4492	1.98 ± 0.100	1.89 ± 0.203	0.2874
Cortical Thickness (mm)	0.195 ± 0.010	0.201 ± 0.028	0.5978	0.231 ± 0.015	0.223 ± 0.013	0.2986
Tissue Mineral Density (g/cm^3)	0.830 ± 0.057	0.817 ± 0.057	0.6724	0.907 ± 0.038	0.924 ± 0.048	0.4463

Data are presented as mean ± SD (unpaired t-test KO vs WT for each sex). BV/TV; bone volume/tissue volume.

Table S7. Trabecular and cortical bone parameters of 6-month-old *Cldn12* KO vs. WT mice

	Male			Female		
	**WT**	***Cldn12* KO**	**P- value**	**WT**	***Cldn12* KO**	**P- value**
N	6	6		6	6	
**Trabecular Bone**						
Bone Mineral Density (g/cm^3)	0.112 ± 0.020	0.158 ± 0.048	0.0931	0.096 ± 0.001	0.103 ± 0.014	0.3159
BV/TV (%)	6.95 ± 2.09	11.14 ± 6.39	0.1763	4.27 ± 1.03	4.51 ± 1.38	0.7422
Trabecular Thickness (mm)	0.063 ± 0.002	0.068 ± 0.009	0.1967	0.046 ± 0.010	0.045 ± 0.010	0.8432
Trabecular Separation (mm)	0.463 ± 0.151	0.318 ± 0.064	0.0566	0.667 ± 0.136	0.572 ± 0.085	0.1759
Trabecular Number (1/mm)	1.10 ± 0.306	1.57 ± 0.731	0.1729	0.976 ± 0.356	1.02 ± 0.318	0.8428
**Cortical Bone**						
Bone Volume (mm^3)	1.87 ± 0.189	2.07 ± 0.105	0.0452	1.98 ± 0.202	1.86 ± 0.276	0.4222
Cortical Thickness (mm)	0.199 ± 0.014	0.205 ± 0.012	0.4790	0.230 ± 0.022	0.214 ± 0.017	0.1859
Tissue Mineral Density (g/cm^3)	0.894 ± 0.035	0.905 ± 0.026	0.5787	0.949 ± 0.008	0.950 ± 0.043	0.9549

Data are presented as mean ± SD (unpaired t-test KO vs WT for each sex). BV/TV; bone volume/tissue volume.

Table S8. Transepithelial resistance and ion permeability of Caco-2 cells expressing claudin-12 under a tet-off system

	**- Dox** (n = 12)	**+ Dox** (n = 12)	***P* – value**
**TER (Ω cm^2)**	166 ± 60	199 ± 44	0.1400
**TER (normalized)**	1.00 ± 0.19	1.24 ± 0.19	0.0053
**P_{Na} (normalized)**	1.00 ± 0.18	0.83 ± 0.10	0.0074
**P_{Cl} (normalized)**	1.00 ± 0.22	0.76 ± 0.17	0.0066
**P_{Na}/P_{Cl} (normalized)**	1.00 ± 0.13	1.09 ± 0.12	0.1773
**P_{Ca}/P_{Na} (normalized)**	1.00 ± 0.07	1.00 ± 0.05	0.8888

TER, transepithelial resistance; P_x, permeability to ion x; Dox, doxycycline. Data presented as mean ± SD. *P* – value determined by unpaired t-test. All data normalized to without Dox treatment for each day of experiments where indicated.

Table S9. Transepithelial resistance and ion permeability of OK cells stably expressing empty vector or claudin-2

	**EV** (n = 6)	**CLDN2** (n = 8)	***P* – value**
**TER (Ω cm^2)**	13.4 ± 3.56	3.01 ± 1.89	0.0012
**P_{Na} ($x10^{-4}$ cm s^{-1})**	1.08 ± 0.32	6.23 ± 3.69	0.0186
**P_{Cl} ($x10^{-4}$ cm s^{-1})**	1.04 ± 0.30	6.18 ± 3.76	0.0200
**P_{Na}/P_{Cl}**	1.04 ± 0.02	1.02 ± 0.03	0.1683
**P_{Ca}/P_{Na}**	2.96 ± 0.05	2.99 ± 0.03	0.2668

TER, transepithelial resistance; P_x, permeability to ion x; EV, empty vector. Data presented as mean ± SD. *P* – value determined by unpaired t-test.

Supplementary Methods

Animals and husbandry

Mice were housed on a 12-hour light/dark cycle with drinking water and chow *ad libitum* (Lab Diet Irradiated Rodent Diet 5053, 4% fat, 0.81% calcium). For all experiments, male and female mice were used. Data presented includes both males and females as sex-specific differences were not found in any analysis except where explicitly indicated. FVB/N, (Taconic Biosciences, Rensselaer, NY), *Cldn2* global KO (MMRRC, Univ. of California, Davis), and *Cldn12* global KO mice were used.

Metabolic cage studies

WT and *Cldn2/12* DKO mice aged 2-3 months, with approximately equal numbers of both genders were placed in metabolic cages for 72 hours. Water and chow (0.6% Calcium) were available *ad libitum*. Body weight was determined at time 0 h. Urine, feces, body weight, chow and water consumed were monitored every 24 hours. After 72 hours, mice were euthanized with a lethal dose of sodium pentobarbital. Blood was collected in lithium heparin-coated tubes and centrifuged at 3500 rpm for 20 minutes at 4°C to collect serum which was then stored at -80°C. Tissues were excised, rinsed in PBS and stored at -80°C.

Urine and serum analysis

Freshly collected blood was analyzed for electrolytes, ionized calcium (iCa), glucose, urea nitrogen (BUN), hematocrit (Hct) and hemoglobin (Hgb) using an i-STAT1 Analyzer (Abaxis, Union City, CA, USA) with a CHEM8+ cartridge. Serum creatinine was measured with Diazyme creatinine kit (Diazyme Laboratories, CA, USA). Urine creatinine was measured with Parameter creatinine kit (R&D systems, Minneapolis, USA). Urine electrolytes were measured by ion chromatography (Dionex Aquion Ion Chromatography System, Thermo Fisher Scientific Inc., Mississauga, ON, Canada) with autosampler. Samples were diluted 1:100 in ddH$_2$O and carried in 4.5 mM Na_2CO_3/1.5 mM $NaHCO_3$ in ddH$_2$O for anion eluent, 20 mM Methanesulfonic acid in ddH$_2$O for cation eluent. Calibration curves were created with serial

dilutions of Dionex five anion and six cation-I standards (Dionex, Thermo Fisher Scientific Inc., Mississauga, ON, Canada). Results were analyzed using Chromeleon 7 Chromatography Data System software (Thermo Scientific). Urine cations were normalized to urine creatinine concentration. FECa was calculated as (urine Ca^{2+} * serum creatinine) / (serum iCa^{2+} * urine creatinine). PTH (Immutopics Mouse Intact PTH 1–84) and 1,25$(OH)_2$-vitamin D were measured by ELISA (Immunodiagnostic Systems Limited, Boldon, UK).

Ca^{2+} bioavailability

Feces was collected from metabolic cage studies and dried at 55°C for 72 hours (Imperial III Incubator, Labline, Mumbai, India). Dried pellets were then ground with mortar and pestle. After mixing the powder, 50 mg was taken and solubilized in 1 ml 0.6 M HCl and rotated for 72 hours. A 1 mL aliquot was diluted 1:50 and total Ca^{2+} measured using ion chromatography as above. Bioavailability is presented as the percentage of Ca^{2+} consumed in the chow ((mg of Ca^{2+} consumed – mg of Ca^{2+} in feces) / mg of Ca^{2+} consumed *100). Ca^{2+} balance was calculated as: (mg Ca^{2+} ingested) – (mg Ca^{2+} in feces) – (mg Ca^{2+} in urine).

Real-time PCR

Total RNA was isolated from frozen tissues using the TRIzol method (Invitrogen, Carlsbad, CA) according to the manufacturer's instructions and treated with DNAse (ThermoScientific, Vilnius, Lithuania). RNA purity and quantity were measured using a Nanodrop 2000 (Thermo Fisher Scientific, Waltham, MA). Five micrograms of RNA were then reverse transcribed using reverse transcriptase (SensiFAST cDNA Synthesis Kit, FroggaBio, CA, USA). A pooled sample of RNA was used to create cDNA with serial dilutions for the standard curve. Quantitative RT-PCR was performed in triplicate on each sample using TaqMan Universal Master Mix II (ThermoFischer Scientific) with specific primers and probes on a QuantStudio 6 Pro Real Time PCR System (ThermoFisher Scientific). Specificity of primer sequences was assessed with NCBI Primer-BLAST. Samples were quantified using the standard curve method. A C_q value of greater than 35 was considered negligible.

Measurement of Ca^{2+} permeability

Fresh intestinal tissue was excised from euthanized mice, rinsed in Kreb's ringer buffer (KRB), linearized, and mounted onto P2407B sliders in P2400 Ussing chambers connected to a VCC multichannel voltage/current clamp (Physiologic Instruments, San Diego, CA). A 1.2 cm long section of tissue immediately distal to the cecum from each animal was excised and cut into four sections to represent proximal colon. One mouse was used for each experiment and represents one data point. Tissue was bathed in KRB as above as a "control" buffer (144 mM Na^+, 1 mM Mg^{2+}, 1.3 mM Ca^{2+}, 2 mM PO_4^-, 3.6 mM K^+, 146 mM Cl^-, pH 7.4). After 15 minutes, a 90 µA current was applied and the recorded voltage change used to determine TER with Ohm's law. The apical buffer was then changed to a low-NaCl isotonic buffer (30 mM Na^+, 1 mM Mg^{2+}, 1.3 mM Ca^{2+}, 2 mM PO^{4-}, 3.6 mM K^+, 32 mM Cl^-, 227 mM mannitol, pH 7.4). The resulting peak change in transepithelial voltage was used to determine the permeability ratio of Na^+ to Cl^- (P_{Na} /P_{Cl}) and absolute permeability to Na^+ and Cl^- using the Goldman-Hodgkin-Katz and simplified Kimizuka-Koketsu equations. The apical solution was then changed back to the control solution until the potential difference stabilized again and TER was again measured. The basolateral buffer was then changed to a phosphate-free isotonic control (140 mM Na^+, 1 mM Mg^{2+}, 1.3 mM Ca^{2+}, 3.6 mM K^+, 146 mM Cl^-, 5 mM mannitol, 3 mM HEPES, pH 7.4) and the apical to a high Ca^{2+} isotonic buffer (1 mM Mg^{2+}, 70 mM Ca^{2+}, 2 mM PO_4^-, 3.6 mM K^+, 146 mM Cl^-, 3 mM HEPES, pH 7.4) and the peak change in potential difference was again measured and used to calculate P_{Ca}/P_{Na} and $P_{Ca}{}^{2+}$. Buffers were then changed to the control in both chambers and TER measured as above.

Permeability to calcium across epithelial monolayers was performed as above on OK cells expressing empty vector or CLDN2 in pcDNA3.1+ (Invitrogen). Briefly, $1x10^5$ cells were seeded onto 1.12 cm^2 Snapwell inserts (Corning, NY) in DMEM/F-12 medium, supplemented with 10% FBS and 5% penicillin streptomycin glutamine. After 7 days, P_{Ca} was determined as above. After each experiment, cells were removed from the membrane with trypsin and the experiment repeated on the empty filter and these values were subtracted from results with cells. Caco-2 cells were cultured in DMEM medium, supplemented with 10% FBS and 5% penicillin streptomycin glutamine.

Micro-Computed Tomography

Bones were fixed in 4% PFA and stored at 4°C prior to analysis. The right tibial metaphasis was scanned at a resolution of 18 μm using a Skyscan 1176 micro-computed tomography (μCT) imager (Skyscan NV, Kontich, Belgium). Voltage was set to 45 kV, current was set to 555 μA, and low photon energies reduced using a 0.5 mm Al filter, at a 0.5º rotation step. The origin of trabecular bone was taken as the bridging of the metaphyseal growth plate. The region of interest spanned 100 slices starting 20 slices below the landmark. Analysis was conducted using CT-analyser (version 1.14.4.1, Bruker). Representative images were reconstructed using CTVol (Bruker).

Immunofluorescence on Caco-2 cells

Cells were grown to confluence then washed with PBS with 1 mM $CaCl_2$ and 1 mM $MgCl_2$ (pH 7.4), fixed on ice with 4% paraformaldehyde and then quenched with 5% glycine in PBS. Cells were blocked with 5% milk in PBS with 0.2% Triton X-100 for 1 hour with gentle shaking. Cells were then incubated in primary antibody for 1 hour, rinsed with PBS and incubated with secondary antibody for 1 hour. The cover slips were then mounted on a microscope slide with DAKO and visualized at least 24 h later.

Immunoblot

Tissue or cells were lysed in RIPA buffer (50 mM Tris, 150 mM NaCl, 1 mM EDTA, 1% Triton-X, 1% SDS, 1% NP-40, pH 7.4) with 1:100 0.1 M PMSF (Thermo Scientific, Rockford, IL), and 1:100 protease inhibitor cocktail (Calbiochem, San Diego, CA). Protein content was measured using Pierce 660 nm Protein Assay Reagent (ThermoFisher Scientific). 150 μg of tissue protein or 50 μg protein from cell culture was run on 10% SDS-PAGE, electrotransferred to PVDF (Merck Millipore, Burlington, MA) and blocked overnight in TBST with 5% milk. The blots were probed with primary antibody overnight at 4 °C then secondary antibody for 1 hour at room temperature and visualized using Immobilon Crescendo Western HRP substrate (Sigma-Aldrich, Canada) and a ChemiDoc Touch imaging system (Bio-Rad).

Co-immunoprecipitation

Cells were lysed in IPEB buffer (10 mM Tris-HCl, 1% NP-40, 5 mM EDTA, 0.15 M NaCl, pH 7.5) containing 1:100 0.1 M PMSF in 100% ethanol (Thermo Scientific, Rockford, IL), and 1:100 protease inhibitor cocktail (Calbiochem, San Diego, CA) and an aliquot was saved.

Immunohistochemistry and X-gal staining

Colon and kidney tissue was fixed in 4% PFA for 30 min, cryoprotected in 30% sucrose and frozen. Thereafter, tissue was sectioned on a cryomicrotome and stained with X-gal BetaBlue staining solution according to the manufacturer's instructions (Merck, 71074). Tissue was subjected to heat-induced antigen retrieval by boiling in Tris-EGTA (TEG, 10 mM Tris, 0.5 mM EGTA, pH 9.0), incubated in 0.6% H_2O_2 and 50 mM NH_4Cl in PBS and then probed with primary rabbit anti-CLDN2 antibodies (#51-6100, Invitrogen) in 0.1% Triton-X in PBS overnight at 4 °C and then incubated with secondary antibodies. Sections were visualized with DAB^+ Substrate Chromogen System (K3467, DakoCytomation).
